# Smart PEG-Block-PLA/PLA Nanosystems: Impact of the Characteristics of the Polymer Blend on the Redox Responsiveness

**DOI:** 10.3390/ma16020539

**Published:** 2023-01-05

**Authors:** Louise Van Gheluwe, Stephanie David, Eric Buchy, Igor Chourpa, Emilie Munnier

**Affiliations:** 1 EA 6295 Nanomédicaments et Nanosondes, Université de Tours, Faculté de Pharmacie, 31 Avenue Monge, 37200 Tours, France; 2 Laboratoires Eriger, 33 rue Augustin Fresnel, 37170 Chambray les Tours, France

**Keywords:** redox-responsive disulfide bond, mPEG-SS-PLA/PLA blend, smart drug delivery system (SDDS), triggered release

## Abstract

Nanocarriers (NCs) were designed from three polymer blends (B1, B2 and B3) and investigated as smart drug delivery systems (SDDS). The blends are composed of a “smart” copolymer, where methoxy poly(ethylene glycol) and poly(lactic acid) are connected via a redox-responsive disulfide bond (mPEG-SS-PLA), and of a “conventional” polymer, poly(lactic acid) (PLA). They differ by mPEG-SS-PLA/PLA ratio and PLA molecular weight. Nanoprecipitation was used to prepare NCs. Three concentrations were tested, and fluorescent dye Nile red (NR) was used as a model payload. The results show that the characteristics of the NCs, such as size and drug release kinetics, are influenced by the type of blend and the concentration used during the nanoprecipitation process. The more redox-responsive blend was B2 (ratio 1:3, PLA 5 kDa) at 16 mg/mL: the quantity of NR released was tripled upon 24 h of incubation in a reducing medium. This study reveals that the amount of disulfide bonds present in a NC is not the only parameter to be considered to design an SDDS. The stability of the SDDS in a presumably non-stimulating environment is also important to limit uncontrolled release during storage or in the body before the biological target is reached.

## 1. Introduction

The concept of smart drug delivery systems (SDDS) has been created to control the delivery and the release of active ingredients into action site. The drug payload of stimuli-responsive SDDS will be released when a biological or physical stimulus occurs, which needs to be specific to the application. For example, temperature-responsive, pH-responsive or redox-responsive nanocarriers (NCs) are the most widely described smart systems for biomedical uses [1]. The preparation of such SDDS is typically based on using stimuli-responsive polymers. For temperature- or pH-responsive systems, the stimulus will lead to a conformational or a structural modification in the material. The modifications are governed by the transition in the temperature below or above the polymer’s lower critical solution temperature or the ionization of basic or acidic on the polymer chains. Unlike temperature and pH-responsive systems, the production of redox-responsive systems often requires sophisticated chemical modification, notably the insertion of a reduction-sensitive linker such as the well-known disulfide bonds (-SS-). The disulfide bond insertion, either between two polymers [2,3], between the polymer and the drug [4] or in each repeating unit of a polymer [5,6], is time- and money-consuming. Indeed, during the synthesis of stimuli-responsive polymers, several purification and characterization steps are necessary to obtain a pure smart product.

mPEG-*block*-PLA-based nanocarriers have already demonstrated their interest in the encapsulation of nucleic acids, pharmaceutical or cosmetic active ingredients [7,8,9,10,11]. In the formulation process of NCs, many parameters may cause crucial changes in the physi-co-chemical characteristics such as morphology, size and drug encapsulation efficiency (EE) [12,13,14,15]. For example, it was described that the NCs’ morphology depends on the molecular weights of the polymer blocks [15]. Dong and co-workers showed that the mPEG-*b*-PLA nanocarrier size increased when the polymer amount in the organic solvent was increased [14]. Concerning mPEG-*b*-PLA/PLA polymer blends, it was observed that the size of NPs decreased when mPEG-*b*-PLA content increased [16]. Redox-responsive mPEG-*b*-PLA NCs containing disulfide bonds between the polymers were developed to construct drug delivery systems for cancer therapy [17,18]. Nevertheless, few studies have focused on the impact of the material and preparation parameters on the stimuli-responsiveness of smart nanocarriers.

Lately, a method to synthesize a redox-responsive blend of mPEG-b-PLA/PLA polymers was developed in our laboratory [19]. Briefly, a blend of redox-responsive mPEG-SS-PLA copolymer and a non-responsive PLA polymer (ratio 1:2) was synthesized in three steps and used for retinol encapsulation. This study has notably shown that it is possible to design a redox-responsive system with a restricted amount of reactive materials (≈33%), minimizing the cost of the production of the SDDS. Moreover, these nanocarriers are suitable for skin application. Using this synthesis strategy, it is possible to tune the molecular weights of PLA blocks.

Herein, the number of disulfide bonds is linked to the polymer concentration and will most certainly have an impact on the sensitivity of the system to the redox stimulus. The objective of the present study was thus to investigate the impact of the polymeric material, i.e., the PLA molecular weight, the mPEG-SS-PLA/PLA molar ratio as well as the blend concentration, on the main physicochemical characteristics of the NCs, particularly on their sensitivity to the redox stimulus. By modifying conditions of the ring-opening polymerization, mPEG-SS-PLA/PLA copolymers were synthesized with different lengths of PLA chains and PLA/PEG ratios. Then, using three different mPEG-SS-PLA/PLA blends, several NCs were prepared by the nanoprecipitation method. This method enables the spontaneous particle formation without surfactants and with low energy consumption [20,21] and thus can be easily transposed on an industrial scale. Environment-sensitive fluorophore Nile red (NR) was encapsulated in these nanocarriers, and the effect of GSH concentrations on the stimuli-responsive release of the NR was investigated in vitro.

## 2. Materials and Methods

### 2.1. Chemicals

Dichloromethane, methanol, anhydrous toluene, anhydrous acetic acid, tetrahydrofuran, stannous 2-ethylhexanoate, 2-mercaptoethanol, 2,2′-dithiobis(5-nitropyridine), *O*-[2-(3-mercaptopropionylamino)ethyl]-*O*′-methylpolyethylene glycol (PEG-thiol, 5 kDa), 3,6-dimethyl-1,4-dioxane-2,5-dione (d,l-lactide) and Nile red were purchased from Sigma-Aldrich (St Quentin-Fallavier, France). Dialysis membrane (regenerated cellulose, molecular weight cut-off MWCO 2 kDa) was obtained from BioValley (Marne La Vallée, France). l-Glutathione reduced (GSH) was provided by Fisher Scientific (Illkirch, France). Ultrapure water was produced by the Milli-Q system from Millipore (Paris, France).

### 2.2. Synthesis and Characterization of PEG-b-PLA/PLA Redox-Responsive Blend

The first two steps have been described in a previous study [19]. Ethanol, 2-[(5-nitro-2-pyridinyl)dithio]- (abbreviated as compound **1**) was first synthesized. Briefly, 2,2′-dithiobis(5-nitropyridine) was solubilized in dichloromethane. A methanolic solution of 2-mercaptoethanol was prepared and added to the blend. At last, anhydrous acetic acid was added. The solution turned yellow and was allowed to stir for 24 h. Solvents were removed under vacuum and compound **1** was purified by flash chromatography.

The second step corresponds to the synthesis of disulfide PEG (abbreviated as P1). Briefly, PEG-thiol, compound **1** and anhydrous acetic acid were solubilized in methanol and allowed to stir for 30 h. The reaction was monitored using a UV–Visible spectrophotometer (Genesys 10S, Thermo Fisher Scientific, Les Ulis, France) by measuring the absorbance of thionitropyridine (387 nm) released in the reaction medium (yellow color). The solvent was subsequently evaporated in vacuum and the residue was purified by dialysis (water, MWCO 2 kDa, regenerated cellulose). In the end, the product was freeze-dried and a white powder was obtained and stored at −20 °C.

The last step corresponds to the synthesis of redox-responsive copolymer (abbreviated as P2). To obtain different lengths of PLA blocks, the amount of d,l-lactide and the time of reaction differed between blends. Briefly, appropriate amounts of pure d,l-lactide and P1 were solubilized with anhydrous toluene. The blend was degassed by bubbling nitrogen for 20 min before adding the stannous 2-ethyl-hexanoate. The reaction blend was refluxed in an oil bath (120 °C) for an appropriate time. At the appropriate time, the reaction was stopped by removing the toluene under reduced pressure. The residue was solubilized in THF, and size exclusion chromatography purification (Sephadex LH-20, THF) was performed. Purification of the P2 polymers produced from the residual lactide monomer was monitored by infrared spectroscopy (Bruker Vector 22, ATR mode). After collection and concentration of the product under vacuum, the polymers were solubilized in a minimum of THF, then precipitated in cold water. After THF evaporation, the polymers were lyophilized and a white powder was obtained and stored at −20°C.

Characterizations: For each step, ^1^H NMR spectra were recorded with a Bruker 300 MHz NMR spectrometer at 25 °C with CDCl_3_ as solvent. ^1^H NMR (300 MHz, CDCl_3_, ppm): δ 1.57 (m, 3H), 2.58 (t, 2H, J = 7.2 Hz), 2.89 (t, 2H, J = 6.7 Hz), 2.97 (t, 2H, J = 7.1 Hz), 3.37 (s, 3H), 3.40 (t, 2H, J = 4.9 Hz), 3.64 (m, 4H), 3.87 (t, 2H, J = 4.9 Hz), 4.35 (q, 1H, J = 7.0 Hz), 4.38 (t, 2H, J = 6.6 Hz), 5.18 (m, 1H).

### 2.3. Preparation and Characterization of Polymer Nanocarriers

Preparation: With each polymeric blend, polymer nanocarriers labeled with Nile red (NR-NCs) were prepared in triplicate by nanoprecipitation method. Briefly, 0.6 mL of acetone solution composed of 4, 10 or 16 mg/mL of polymer blend and 13 µg/mL of NR was added dropwise to 2 mL of deionized water using a syringe pump (KR Analytical, Sandbach, UK) with a flow rate of 0.3 mL/min and under moderate stirring. Nanocarriers formed instantly. Acetone was evaporated overnight under stirring at room temperature. The ensuing suspensions were filtered through a 0.45 µm membrane to eliminate Nile red in excess. The procedures were carried out in the dark to preserve the fluorescence of the label.

Hydrodynamic diameter and zeta potential: The mean hydrodynamic diameter (DH) and the polydispersity index (PdI) of NCs were measured in aqueous suspensions (dilution 1/10 in ultrapure water) by photon correlation spectroscopy (or dynamic light scattering, DLS) using a Zetasizer^®^ apparatus (Malvern Panalytical, Malvern, UK) with a He-Ne laser (633 nm, scatter angle 173°). The measurement was performed three times at 25 °C. The DLS results are shown in intensity mode. The zeta potential of the particles was measured on the same samples with the same equipment using appropriate cells.

TEM analysis: The nanocarriers’ aqueous suspensions were deposited on carbon film-covered copper grids and then negatively stained. The TEM grids were first plasma-treated in advance, in order to render the carbon support film hydrophilic and improve sample adsorption and spreading, using an ELMO© glow discharge system (Cordouan Technologies, Pessac, France). Then, 5 to 10 µL of the sample suspension was deposited onto a grid. Excess liquid was blotted off with filter paper after 1–2 min of incubation. For staining, three ≈10 µL drops of 1.5% (*w*/*v*) aqueous uranyl acetate solution were successively deposited on the grid and then blotted off, after which the grid was allowed to dry under ambient air. The negatively stained samples were then imaged at 120 kV using a JEOL (Tokyo, Japan) JEM-1400 Plus transmission electron microscope equipped with a GATAN (Pleasanton, CA, USA) OneView CMOS camera.

Cryo-TEM analysis: 5 µL of nanocarriers’ aqueous suspensions was deposited on C-Flat holey carbon grids (CF-1.2/1.33-3Cu-T-50, Protochips, Morrisville, NC, USA) and vitrified with a Vitrobot Mark IV set at 4 °C, 100% humidity (Thermo Fischer, Waltham, MA, USA). Grids were then transferred onto a cryo holder (Cryo-Holder Fischione model 2550, Fischione Instruments, Export, PA, USA) before observations under a transmission electron microscope (JEOL 1400 Plus, Tokyo, Japan).

Nile red contents: The absorbance of the suspensions was measured in a quartz cell (1 cm) placed in a Genesys 10S (Thermo Scientific, France) UV–Vis spectrophotometer. From the absorbance values, Nile red concentration was determined according to a standard calibration curve made in ethanol. The theoretical encapsulation efficiency (EE) was calculated by the equation:

Encapsulation efficiency (%) = Determined weight of Nile red in the nanocarriers/Initial weight of Nile red placed in the reaction medium

### 2.4. Response to the Redox Stimulus of Copolymer-Based Nanocarriers

The redox-mediated response to glutathione (GSH) was evaluated in vitro, using NR-NCs (dilution 1:10 in ultrapure water). Nile red was chosen because it is a fluorescent label sensitive to the chemical environment. First, 0.5 mL of diluted suspension was combined with 0.5 mL of GSH solution (20 mM) or 0.5 mL of diluted nitric acid solution to ensure the same pH. Each tube was shaken at 37 °C over a 24 h period. NR fluorescence was measured after 0 h, 2 h, 4 h, 8 h and 24 h of shaking, using a spectrofluorometer (A Hitachi F-4500). The shape of the fluorescence did not change during the release study; only the intensity of fluorescence varied. The fluorescence excitation wavelength was 535 nm, and the emission fluorescence was recorded within the 555–700 nm spectral range. To study NR release after GSH treatment, the following formula was used:

% of NR released at t = (Fluorescence intensity t_0_ − Fluorescence intensity  t)/Fluorescence intensity t_0_ × 100

The stimulus–response factor was calculated as follows:

% of NR released at 24 h in GSH solution/% of NR released at 24 h in  diluted nitric acid solution × 100

## 3. Results

### 3.1. Synthesis and Characterization of Redox-Responsive PEG-Block-PLA/PLA Blends

**Figure 1:**
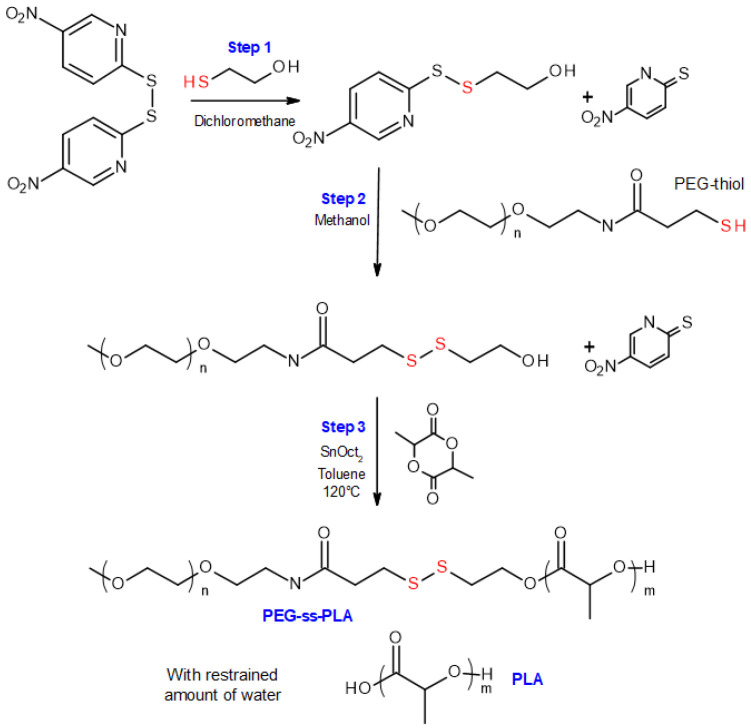
Synthesis scheme of the amphiphilic redox-responsive blends [19].

Three blends of polymers were synthesized by modifying the ingredients’ concentration or the reaction time of the third step of the synthesis developed in the lab, shown in Figure 1 [19].

**Figure 2:**
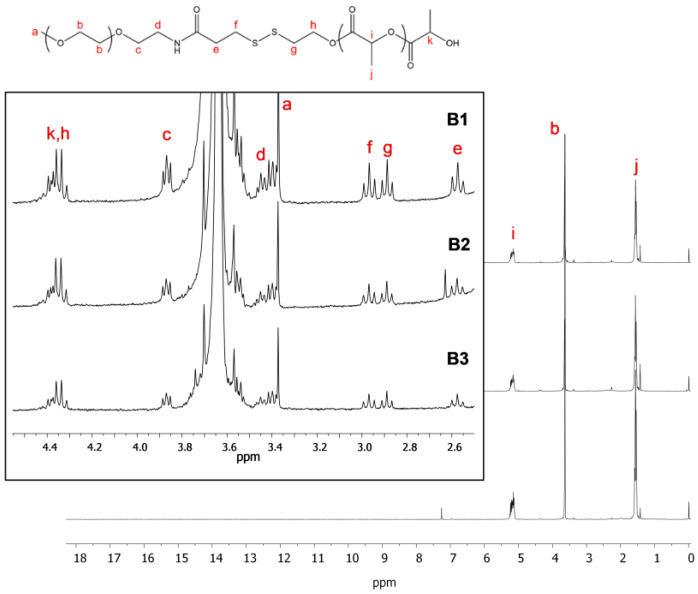
^1^H NRM spectrum of blends 1, 2 and 3 (step 3, CDCl_3_, 25 °C).

After the last step, corresponding to the polymerization of the lactide by ring-opening polymerization, two compounds are expected: mPEG-SS-PLA (P2a), polymerized from the hydroxyl functional group at the end of the modified PEG P1, and PLA (P2b), polymerized from traces of water. These three blends (abbreviated as B1, B2 and B3) differ in the length of the polymers which compose them as well as in the mass ratio between stimuli-sensitive polymer and conventional polymer (Table 1). For each blend, the polymer structure was confirmed by ^1^H NMR, as demonstrated in Figure 2.

Signals located at 5.18 ppm and 1.57 ppm were assigned to the methine (-CH, i) and methyl (-CH_3_, j) in the PLA polymer, respectively. Importantly, the signal located at 4.4 ppm was attributed to the methine end-group of PLA polymer (-CH, k). Methylene protons in the PEG unit were assigned at 3.68 ppm (b). After polymerization of the lactide, the methylene protons signal (-CH_2_-, h) of P1, linked to the hydroxyl group, shifted from 3.46 to 4.4 ppm. This signal overlapped with the signal k corresponding to the methine end-group of PLA polymer. If the end product only corresponded to the copolymer P2a, then the integration at 4.4 ppm was assumed to be 3 (=3H). Herein, the integration was 4.86, 6.02 and 5.09 for blends 1, 2 and 3, respectively. It corresponded to 2H of methylene protons (-CH_2_-, h) and 2.86H, 4.02H and 3.09H, respectively, associated with PLA end-group methine protons (-CH, k). As described before [19], among the k protons, 1H corresponds to the PLA end-group methide proton of P2a, and the rest correspond to the PLA end-group methide protons of PLA polymerized with water traces (P2b). Therefore, different blends of copolymer P2a (mPEG-SS-PLA) and polymer P2b (PLA) were produced with an approximate ratio of 1:2 (blends 1 and 3), and an approximate ratio of 1:3 for blend 2.

PLA molecular weight could be determined using PLA and PEG integrations, notably with methine (-CH, i) and methylene (-CH_2_-, f or g) protons of the lactate unit of PLA and modified PEG, respectively. For example, for blend B2, the “g” signal integrates for 2H and the “i” signal integrates for 263H. One repeating unit of PLA corresponds to 1H “i” (-CH, i) with a molecular weight of 72 g/mol. So, for 1 mole of PEG disulfide, the molecular weight of all repeating units of PLA is: 263H × 72 g/mol ≈ 19,000 g/mol (or 19 kDa). For blend 2, given the ratio mPEG-SS-PLA/PLA of 1:3, four PLA blocks are present, and therefore, the average PLA molecular weight of each block is close to 19/4 = 5 kDa. The same strategy was employed to assess the molecular weight of PLA blocks of other blends. The resulting blends are presented in Table 1. To be able to explore the importance of the characteristics of the polymer blend in the stimuli-responsiveness of the resulting nanocarriers, we chose not to purify it further.

Blends 1 and 2, showing very close polymer sizes, are compared to investigate the impact of polymer ratio on the characteristics of the nanocarriers developed. Blends 1 and 3, showing the same ratio, are compared to investigate the impact of PLA molecular weight on the characteristics of the nanocarriers developed.

### 3.2. Nanocarriers Physicochemical Characteristics

**Figure 3:**
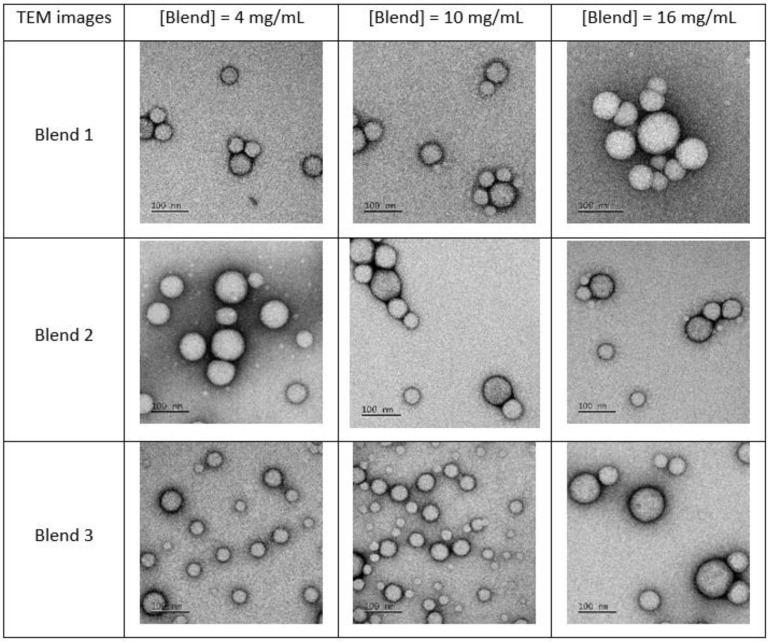
Typical TEM images of nanocarriers obtained from blends 1, 2 and 3, with increasing concentrations.

The nanoprecipitation method was used to produce mPEG-SS-PLA/PLA nanocarriers. This method is one of the most popular methods for the manufacture of polymer NCs for pharmaceutical applications [12,22]. In this method, polymer and hydrophobic active molecules dissolved in a water-miscible solvent are added to an aqueous non-solvent phase under stirring, resulting in the formation of NCs by precipitation. Herein, the NCs were formed by the precipitation of polymer as a result of diffusional exchange of solvent (acetone) and non-solvent (water). Since the organic solvent acetone is miscible to water, the solvent in organic phase migrates spontaneously toward the water phase once the polymer solution in organic solvent is introduced into water. A sudden change in the solvent composition around the polymer molecules finally induces aggregation of hydrophobic blocks of polymer and, subsequently, nanocarrier formation. As it can be seen in TEM images (Figure 3), Nile red-loaded nanocarriers (NR-NCs) have a spherical morphology, regardless of blend or concentration used.

Given that the polymeric material of each blend is different, i.e., smart/conventional polymer ratio and molecular weight of the hydrophobic polymer blocks, different types of nanosystems could have been expected, such as nanomicelles or polymersomes. Indeed, the ratio of the hydrophilic part to the total mass of a copolymer (fPEG value) is determined for the formation of the polymersomes, a fPEG value between 25% and 40% being optimal [23,24]. Herein, for blend 3 (fPEG value of 31% in mPEG-SS-PLA), the hydrophobic, long and bulky segment of PLA should tend to form polymersomes with a bilayer surface. For blends 1 and 2, the structure should be in favor of a core–shell structure where the core is constituted of the PLA blocks while the outer shell is composed of the hydrophilic PEG blocks [20,24,25]. Nevertheless, TEM results (Figure 3) and Cryo-TEM results (Supplementary Information, Table S1) obtained did not allow us to highlight structural differences between the NCs: they seem to be similar in shape, independently from blend type and concentration. This is surely explained by the presence of the “conventional” polymer PLA in the blend, changing the fPEG value. In a general way, NCs’ characteristics (e.g., the kinetic profiles of drug release) may differ depending on their structure [24,26].

**Figure 4:**
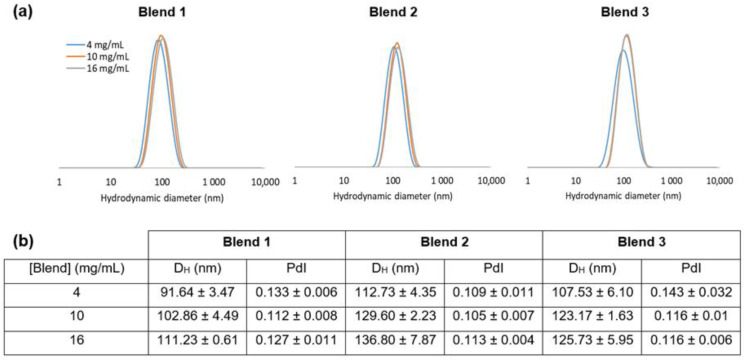
Characteristics of Nile red-labeled nanocarriers: (**a**) hydrodynamic diameter (D_H_) distribution plots; (**b**) associated values of hydrodynamic diameter (D_H_) and polydispersity index (PdI).

Even if TEM images seem to show that the samples are composed of particles of various sizes, the mean hydrodynamic diameter (D_H_) of nanocarriers is between 90 and 140 nm, and the size distribution analysis is in favor of monodisperse samples with a polydispersity index (PdI) close to 0.1 (Figure 4). The D_H_ and PdI values of the three tested blends did not vary significantly over 1 year, indicating the physical stability of the suspension (Supplementary Information, Table S2). The D_H_ values increased by about 20 nm as the concentration of the polymer blend during the nanoprecipitation process was quadrupled. During the nanoprecipitation process, the diffusion rate is expected to be governed largely by the polymer–solvent, polymer–nonsolvent and solvent–nonsolvent interactions as well as the viscosity of polymer solution. The change in viscosity of the organic phase affects the diffusion rate between organic phase and aqueous coagulation phase. Higher concentrations of polymer in organic phase may induce more viscous media and slow diffusion, making it more difficult to disperse organic phase into aqueous phase. Consequently, the organic domains might be solidified before they are dispersed into smaller ones, and bigger particles were formed [12,13,14,16,27,28]. In the present case, a higher concentration of the polymer in the organic phase provides bigger NCs with, certainly, a greater number of redox-responsive bonds per particle.

Furthermore, it seems that the size of NCs decreased as the global PEG wt content in the mPEG-SS-PLA/PLA blend increased. For example, with 10 mg/mL, the D_H_ was 103 nm for B1-based NCs (fPEG of blend = 31%), 130 nm for B2-based NCs (fPEG of blend = 20%) and 123 nm for B3-based NCs (fPEG of blend = 13%). This was also observed by Gref et al., who investigated the effects of the blend ratio on the physicochemical and pharmaceutical properties of NC made of blends of PLA and mPEG-PLA. The particle size was found to progressively decrease upon increasing the PEG weight fraction, possibly because of the amphiphilic nature of these copolymers, reducing the interfacial tension between the aqueous and the organic phases [28]. A similar phenomenon has also been described by Dong et al. [16]. Paclitaxel-loaded nanocarriers were prepared by the nanoprecipitation method from mPEG-PLA/PLA blends at various blend ratios. Increasing the mPEG-PLA fraction in the blends from 0 to 100% resulted in the particle size decrease from 230.6 to 74.8 nm.

The zeta potentials of the nanocarriers were all negative, which is usual for this type of copolymer-based nanocarrier [29,30,31]. The mean zeta potential of all the nanocarriers prepared was −28 ± 5 mV. This mean did not change significantly after three months at 4 °C (−30 ± 2 mV). This negative zeta potential, associated with the steric hindrance of PEG chains, ensures the stability of the suspensions.

Nile red was encapsulated in the nanosystems as a label to be able to determine the responsiveness of the different NCs to the stimulus. It was dissolved, used at the same concentration for all the NCs’ preparation. In these conditions, the encapsulation efficiency (EE) depended on the concentration of the polymer used for the nanoprecipitation. Indeed, the EE was close to 25% wt/wt for each blend when they were used at a concentration of 4 mg/mL (24 ± 1 %, 25 ± 1 % and 27 ± 3 % for B1, B2 and B3, respectively). When the concentration increased to 10 mg/mL, the encapsulation efficiency was multiplied by ≈3.5 (86 ± 3 %, 87 ± 3 % and 89 ± 4 % for B1, B2 and B3, respectively). The EE was not enhanced when the concentration used to prepare the NCs was increased to 16 mg/mL (88 ± 2 %, 88 ± 1 % and 89 ± 3 % for B1, B2 and B3, respectively).

### 3.3. Nile Red Release from the Nanocarriers in a Reductive Medium

**Figure 5:**
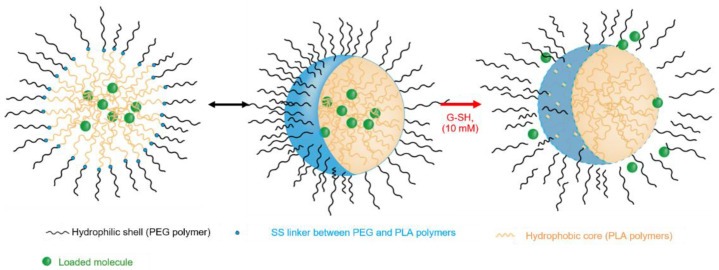
Schematic representation of release of the encapsulated molecule from the mPEG-SS-PLA/PLA core–shell nanocarriers.

Glutathione (GSH) is a detoxification molecule naturally present in human tissues. It shows a reactive thiol function. In cytosol and nuclei of human cells, the concentration of GSH can reach 10 mM, while outside the cell the concentration, the concentration is rather around 2–10 µM [32,33,34]. The insertion of redox-sensitive disulfide linkage into the copolymer-based NCs is likely to accelerate the release of the payload in GSH-rich environment (Figure 5) [17].

In order to follow their behavior in a reducing medium in vitro, NCs were loaded with ≈0.1 % w/w of the solvatochromic fluorophore Nile red (NR) [35]. The NR fluorescence spectra (λ_ex_ = 535 nm) were obtained from aqueous suspensions of NCs, exposed or not to GSH, at different times of incubation, at 37 °C. The NR fluorescence is known to depend on its chemical environment. The NR release from the hydrophobic core of nanocarriers to the surrounding aqueous environment will lead to a dramatic decrease in its fluorescence yield [36]. The NR fraction released from mPEG-SS-PLA/PLA was calculated as described in Section 2, and release kinetics curves were established.

#### 3.3.1. Impact of the mPEG-SS-PLA/PLA Ratio on the Physico-Chemical Characteristics of NCs

**Figure 6:**
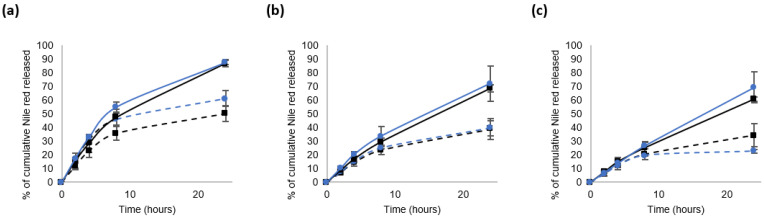
In vitro release of Nile red from the nanocarriers at 37 °C in water. The blends were used at three concentrations: 4 mg/mL (**a**), 10 mg/mL (**b**) or 16 mg/mL (**c**). The curves are encoded as follows: blend 1 (black square) or blend 2 (blue round); without GSH (dashed lines) and in the presence of 10 mM GSH (continuous lines).

The Figure 6 compiles the results obtained for blends 1 and 2, notably to investigate the impact of the mPEG-SS-PLA/PLA ratio on the payload release kinetics.

Whatever the blend or the concentrations tested, the cumulative release of NR was higher in the GSH-enriched environment compared to the control (0 mM GSH). With an increasing concentration of mPEG-SS-PLA/PLA blends for NCs’ development, the overall release of NR decreased. In view of what has been stated above, it can be explained as follows: at a higher blend concentration during the nanoprecipitation process, more polymer chains are involved in the formation of one nanocarrier. Consequently, the affinity between the hydrophobic core and the hydrophobic encapsulated molecule, such as Nile red, increases, which hinders the NR release. In a GSH-enriched medium, this effect is less strong because the nanocarrier sensitivity to glutathione is increased.

For the blend concentrations of 4 or 10 mg/mL, no significant difference was observed regarding the release profiles of Nile red of B1- or B2-based NCs; after 24 h of treatment, the stimulus–response factors were 1.6 and 1.8, respectively. At 16 mg/mL and after 24 h of treatment, a significant difference emerged between B1 and B2. In a control solution, without GSH, the cumulative release of NR was about 34% for B1 NCs and 23% for B2 NCs. In a GSH-enriched solution, the cumulative release of NR was about 61% for B1 NCs and 69% for B2 NCs. Then, the stimulus–response factor was 1.8 for B1 and 3.1 for B2. Therefore, at 16 mg/mL and after 24 h of reaction, the NCs produced from B2 have better sensitivity to a reducing medium compared to those made of B1. For the same mass concentration, it is supposed that more redox-responsive disulfide bonds were involved in the formation of a NC based on B1 than on B2. Therefore, the sensitivity of the NCs to a reducing medium does not depend only on the quantity of sensitive linkers, but also on NCs’ stability in the control solution.

The release kinetics can then be modulated by adjusting the blend ratio. Using the blend B2 (ratio mPEG-SS-PLA/PLA 1:3) at 16 mg/mL makes it possible to produce more stable NCs in the control solution with a better stimulus–response factor than using blend B1 (ratio 1:2). The more PLA in the blend, the slower the release of the drug will be in a non-stimulating environment, favoring in this study a higher stimulus–response factor. This phenomenon was notably observed for NCs made of non-responsive blend mPEG-PLA/PLA. Indeed, in the study by Dong et al., the drug release was slower from the NCs made of pure PLA NCs than from those made of only mPEG-PLA [16]. The drug release rates from NCs made of polymeric blends (mPEG-PLA/PLA ratio of 75/25, 50/50 and 25/75) were found in between values, adjustable according to the therapeutic needs.

#### 3.3.2. Impact of the Molecular Weight of PLA Blocks on the Physico-Chemical Characteristics of the Nanocarriers

**Figure 7:**
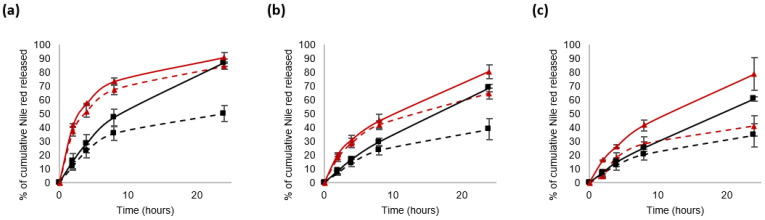
In vitro release of Nile red from the nanocarriers at 37 °C in water. The blends were used at 4 mg/mL (**a**), 10 mg/mL (**b**) or 16 mg/mL (**c**). The curves are encoded as follows: blend 1 (black square) or blend 3 (red triangle); without GSH (dashed lines) and in the presence of 10 mM GSH (continuous lines).

Figure 7 compiles the results obtained for blends 1 and 3, notably to investigate the impact of the molecular weight of PLA blocks on the NCs’ characteristics.

The 24 h treatment with GSH did not affect the NR release from the NCs made of blend 3 (B3) at 4 mg/mL, while at 10 mg/mL and 16 mg/mL, the percentage of cumulative NR released was multiplied by 1.25 and 1.6, respectively. With the NCs made of 16 mg/mL of B3, the NR release in a GSH-enriched medium began to accelerate from 2 h of incubation. Indeed, the cumulative release of NR was 5% in the control solution compared to 16% in the GSH-enriched environment. After 24 h, the stimulus–response factor was 1.6 for B3-based NCs, whereas it was 1.8 for B1-based NCs. Herein, the significant difference between the B1 and B3 blends was the molecular weight of PLA blocks, which is 3–4 kDa for B1 and 11 kDa for B3. This means that for the same mass of each blend, the number of disulfide bonds in the NCs decreases alongside the increase in the PLA length. This could explain the differences in release kinetics observed between B1- and B3-based NCs. It was observed that the redox-responsiveness of B3-based NCs increased with blend concentration. This may be associated with the increase in the quantity of disulfide bonds in a NC but also with better stability of NCs in the control solution, limiting the uncontrolled release of Nile red.

**Figure 8:**
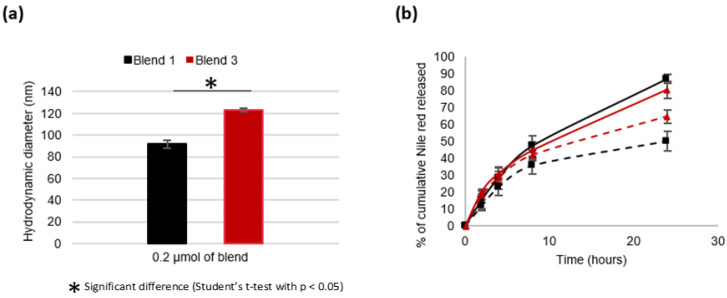
Impact of PLA chain length on the properties and characteristics of nanocarriers. (**a**) Hydrodynamic diameter D_H_. (**b**) Kinetics of the in vitro release of Nile red at 37 °C from the NCs made of 0.2 µmol of blend: B1 (black square) or B3 (red triangle); without GSH (dashed lines); in the presence of 10 mM GSH (continuous line).

Few studies deal with the impact of PLA blocks on nanosystems’ characteristics [29,37]. In order to specifically explore the impact of PLA chain length, NCs made of the same molar concentration of the blend (same number of disulfide bonds, same number of PLA chains but of different length: 3–4 kDa vs. 11 kDa for B1 and B3, respectively) were compared. Thus, the results obtained with 4 mg/mL of B1 and those obtained with 10 mg/mL of B3 were compared (Figure 8) as they correspond to ≈0.2 µmol of each blend.

Nanocarriers developed from 0.2 µmol of the B1 blend possessed a size of approximately 91.6 ± 3.5 nm vs. 123.2 ± 1.6 nm for NCs developed from 0.2 µmol of the B3 blend. As described above, the more PLA there is during nanoprecipitation process, the more viscous the polymer solution and the bigger the NCs [16,27,28].

Regarding the NR release profiles, a significant difference was observed, depending on the GSH treatment and the type of blend used. In a non-stimulating medium, the release of NR is faster when it is initially contained in NCs based on B3 compared to those based on B1. This means that NR is less prone to uncontrolled release from NCs based on 0.2 µmol B1 than those based on 0.2 µmol B3. After a 24 h long stay in the release medium (10 mM GSH), the stimulus–response factors were 1.6 and 1.2 for the NCs made of B1 and B3, respectively. Therefore, the shorter the lengths of PLA, the better the sensitivity of the system to the redox stimulus. Probably, the shorter PLA chains lead to a distribution of the polymer chains in favor of better stability of the NCs and a better accessibility of the disulfide bonds for the reducing agent and/or a rapid destructuring of the nanocarriers once the disulfide bonds have been reduced.

## 4. Conclusions

Using a blend of polymers is an economical, convenient and often-adopted approach to obtain nanocarrier products with desirable properties. Notably, blends of mPEG-PLA and PLA were investigated by different research teams [15,16]. This study demonstrates that with small changes in the polymer blend synthesis scheme we developed, which is easily transferable to industry, nanocarriers with different properties can be prepared. It was observed that blend 2 (mPEG-SS-PLA/PLA, ratio 1:3), at a concentration of 16 mg/mL, made it possible to develop the most stimuli-sensitive nanocarriers. Indeed, after 24 h in a medium rich in glutathione, the stimulus–response factor was 3, i.e., the release of Nile red was multiplied by about 3 times compared to in a control solution, without trigger.

During the nanoprecipitation process, the higher the concentration of the polymer blend, the more disulfide bonds in the nanocarriers, and the more NCs will be susceptible to be redox-responsive. Conversely, the higher the concentration of the polymer blend, the slower the release of NR due to the increasing affinity of the lipophilic label to the hydrophobic core of the NC. The choice of the polymer blend and/or nanoprecipitation parameters is therefore a balance between the sensitivity of the system regarding the trigger (here, glutathione), its stability in a non-stimulable environment and the affinity of the loaded molecule to the polymers.

## Figures and Tables

**Table 1:** Molecular characteristics of each blend.

No. Blend	mPEG-SS-PLA Molecular Weight (kDa)	PLA Molecular Weight (kDa)	Molar Ratio mPEG-SS-PLA/PLA	PEG Content (f_PEG_) in mPEG-SS-PLA	PEG Content (f_PEG_) in Blend	Representative Diagram of the Blends *
1	8 kDa	4 kDa	1:2	63%	31%	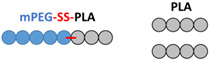
2	10 kDa	5 kDa	1:3	50%	20%	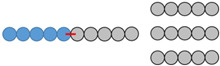
3	16 kDa	11 kDa	1:2	31%	13%	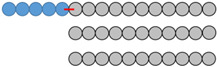

* Each ball corresponds to 1 kDa.

## Data Availability

Data are available upon request to the corresponding author, E. Munnier.

## Supplementary Materials

The following supporting information can be downloaded at: https://www.mdpi.com/article/10.3390/ma16020539/s1, Table S1. Typical Cryo-TEM images of nanocarriers obtained from blend 1, 2 and 3, with a concentration of 16 mg/mL. Table S2. Characteristics of Nile red-labelled nanocarriers (mean values ± SD, n = 3) after 1 year stored at 4 °C.
