# Supplementary material for: Smart PEG-Block-PLA/PLA Nanosystems: Impact of the Characteristics of the Polymer Blend on the Redox Responsiveness
Source: Materials (Basel). 2023 Jan 5;16(2):539. doi: 10.3390/ma16020539 (PMC9864163; doi:10.3390/ma16020539)
Supplement: Supplementary file 1 [file materials-16-00539-s001.zip › materials-1991792-supplementary.pdf]

Supplementary information

Table S1: Typical Cryo-TEM images of nanocarriers obtained from blend 1, 2 and 3, with a concentration of 16 mg/mL.

Cryo-TEM images	[Blend] = 16 mg/mL
Blend 1	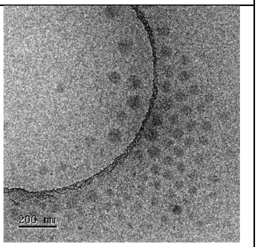
Blend 2	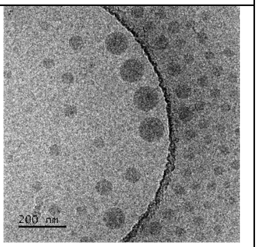
Blend 3	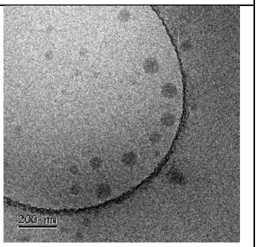

Table S2: Characteristics of Nile red-labelled nanocarriers (mean values ± SD, n=3) after 1 year stored at 4 °C: the hydrodynamic diameter (DH) and PdI.

	Nile red-labelled mPEG-SS-PLA/PLA nanocarriers - **Blend 1**		Nile red-labelled mPEG-SS-PLA/PLA nanocarriers - **Blend 2**		Nile red-labelled mPEG-SS-PLA/PLA nanocarriers - **Blend 3**	
[Blend] (mg/mL)	D_H (nm)	PdI	D_H (nm)	PdI	D_H (nm)	PdI
4	93.21 ± 1.46	0.167 ± 0.036	109.87 ± 2.58	0.116 ± 0.006	100.46 ± 9.93	0.121 ± 0.006
10	95.51 ± 3.82	0.083 ± 0.013	124.70 ± 1.54	0.089 ± 0.010	118.90 ± 0.44	0.106 ± 0.003
16	99.59 ± 3.06	0.079 ± 0.015	131.77 ± 3.33	0.094 ± 0.012	124.97 ± 2.99	0.107 ± 0.011
